# Nucleotide Binding Modes in a Motor Protein Revealed by ^31^P‐ and ^1^H‐Detected MAS Solid‐State NMR Spectroscopy

**DOI:** 10.1002/cbic.201900439

**Published:** 2019-09-30

**Authors:** Thomas Wiegand, Maarten Schledorn, Alexander A. Malär, Riccardo Cadalbert, Alexander Däpp, Laurent Terradot, Beat H. Meier, Anja Böckmann

**Affiliations:** ^1^ Physical Chemistry ETH Zurich Vladimir-Prelog-Weg 1-5/10 8093 Zürich Switzerland; ^2^ Molecular Microbiology and Structural Biochemistry Labex Ecofect UMR 5086 CNRS/Université de Lyon 7 Passage du vercors 69367 Lyon France

**Keywords:** DnaB helicases, fast MAS, hydrogen bonds, nucleotide binding, solid-state NMR spectroscopy

## Abstract

Protein–nucleic acid interactions play important roles not only in energy‐providing reactions, such as ATP hydrolysis, but also in reading, extending, packaging, or repairing genomes. Although they can often be analyzed in detail with X‐ray crystallography, complementary methods are needed to visualize them in complexes, which are not crystalline. Here, we show how solid‐state NMR spectroscopy can detect and classify protein–nucleic interactions through site‐specific ^1^H‐ and ^31^P‐detected spectroscopic methods. The sensitivity of ^1^H chemical‐shift values on noncovalent interactions involved in these molecular recognition processes is exploited allowing us to probe directly the chemical bonding state, an information, which is not directly accessible from an X‐ray structure. We show that these methods can characterize interactions in easy‐to‐prepare sediments of the 708 kDa dodecameric DnaB helicase in complex with ADP:AlF_4_^−^:DNA, and this despite the very challenging size of the complex.

## Graphical abstract

**Protein–nucleotide contacts** in a noncrystalline bacterial DnaB helicase sedimented in the NMR rotor were probed by ^31^P‐ and ^1^H‐detected solid‐state NMR spectroscopy. The sensitivity of ^1^H chemical shifts to hydrogen bonding was explored and used to identify residues involved in nucleotide coordination.

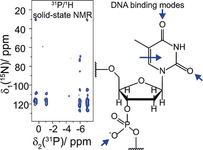

Nucleotide–protein interactions play a central role in two major biological functions: in energy‐providing reactions, where ATP is hydrolyzed to yield energy to motor domains driving reactions^1^, ^2^ and in interactions with RNA or DNA, central in a large variety of biomolecular functions. Binding of nucleotides, such as ATP and DNA, occurs through noncovalent interactions including hydrogen bonds, electrostatic (salt bridges), and van der Waals interactions^3^, ^4^ (the latter also comprising interactions between aromatic rings^5^). These interactions have been typically studied in the past by high‐resolution X‐ray crystallography.^4^, ^6^, ^7^ Still, many of the scenarios described above involve protein complexes, which are difficult to crystallize, and when they do so, might reflect at insufficient resolution to clearly identify interactions. Alternative methods are therefore needed and can be provided through solid‐state NMR spectroscopy, which can access also large biomolecular complexes, and importantly in sample formats where the assemblies are simply sedimented into the NMR rotor.^8^ And indeed, solid‐state NMR spectroscopy has been used to identify residues at protein–RNA interfaces in smaller proteins.^9^, ^10^, ^11^, ^12^

Two approaches are particularly promising to probe nucleotide interactions: phosphorus‐ (^31^P) and proton‐ (^1^H) detected spectroscopy. Distances between ^31^P spins of DNA and ^15^N spins of a protein have been measured by using transferred‐echo, double‐resonance (TEDOR) experiments.^9^ Intermolecular information can also be obtained from ^31^P‐detected, heteronuclear correlation experiments probing the spatial proximity of nucleotide ^31^P and protein ^15^N or ^13^C nuclei.^9^, ^13^ Proton‐detected solid‐state NMR spectroscopy at fast MAS frequencies has emerged in the last years and needs today only a few hundred micrograms of fully protonated protein sample.^14^, ^15^, ^16^, ^17^, ^18^, ^19^, ^20^, ^21^, ^22^, ^23^ Proton chemical‐shift values are highly sensitive to hydrogen bonding^24^, ^25^, ^26^, ^27^ as shown in theoretical,^26^, ^27^ but also in experimental studies.^24^, ^25^, ^28^ Empirical correlations between the ^1^H chemical‐shift values of amide as well as aliphatic protons^29^ and the strength of the hydrogen bond (characterized by the hydrogen bond length) have been established for biological systems.^27^, ^29^, ^30^, ^31^, ^32^ Still, one has to keep in mind that proton chemical shifts can further be influenced by anisotropic neighbor effects, ring current effects,^33^, ^34^, ^35^ and the secondary structure.^36^ This underlines the importance of combination with evidence from ^31^P correlations delivering direct geometric information.

We herein use the dodecameric bacterial DnaB helicase from *Helicobacter pylori* with a molecular weight of 12×59 kDa^37^ as a model to establish approaches to identify protein–nucleic acid interactions in large proteins by solid‐state NMR spectroscopy. DnaB helicases are motor proteins, which coordinate both ATP and DNA:^1^ ATP and a Mg^2+^ cofactor are bound by the Walker A and B motifs as well as the arginine finger (R‐finger) connecting two adjacent subunits of the oligomeric assembly,^38^ whereas DNA binds in the central space of the hexameric proteins to so‐called DNA binding loops. It has been revealed from crystal structures^4^, ^6^, ^39^ that the major coordination partners are Lys and Arg side chains. On the DNA side, important recognition motifs are hydrogen bonds or electrostatic interactions (salt bridges)^40^, ^41^, ^42^, ^43^ involving the DNA phosphate groups as hydrogen‐bond acceptors, but also the ribose or the base moieties.^4^ We previously established that in DnaB from *H. pylori*, the ATP hydrolysis transition state, mimicked by ADP:AlF_4_^−^, preorganizes the helicase for binding single‐stranded DNA to the C‐terminal domain (CTD).^44^ Upon DNA binding, a large fraction of the protein covering the DNA binding loops stiffens, among them 357R and 373K, potentially involved in DNA binding.^44^ We formerly sequentially assigned 70 % of the N‐terminal domain (NTD)^45^ and approximately 60 % of the CTD (311 residues) of DnaB (^15^N, ^13^Cα, and ^13^Cβ, BMRB accession number 27 879) by using ^13^C detection. Also, we previously observed that the NTD is not observed in the DnaB:ADP: AlF_4_^−^:DNA sample, likely due to dynamics, which reduces the number of observed spins to the CTD.^44^

We here describe the ^1^H resonance assignment of the protein (≈45 % of the CTD, see BMRB accession number 27 879 and Table S1 in the Supporting Information), and record ^31^P–^15^N/^13^C heteronuclear correlation spectra. We show how these data can be used to reveal nucleotide–protein interactions, and to determine binding modes, in particular whether DNA coordinates to DnaB through the phosphate groups or base edges. We identify key residues involved in ATP and DNA binding located in the Walker A motif and the DNA binding loops, and compare them to data described for DnaB from *Bacillus stearothermophilus* (*Bst*DnaB),^7^ where a crystal structure is available of the GDP:AlF_4_^−^:DNA‐bound state.

H^N^‐ and H^A^‐detected two‐dimensional hNH and hCH spectra of DnaB:ADP:AlF_4_^−^:DNA recorded at a MAS frequency of 110 kHz show a significant number of resolved signals, and are shown in Figures 1 A and 2 B. For resonance assignments, 3D hCANH, hNCAH, hCAcoNH, and hNcoCAH experiments were recorded. The assignment strategy is illustrated in Figure 1 B. It allows to walk along the protein backbone and delivers the H^N^, H^A^, Cα, and N chemical shifts. Each correlation between a pair of nuclei appears in two independent experiments, as already proven central in ^13^C‐detected experiments.^46^ Figure 1 C shows 2D planes of the 3D spectra illustrating the assignment strategy at the example of residues 448G to 450T (for a second example see Figure S1). Around 45 % of the CTD H^N^ and H^A^ resonances (142 and 139 resonances, respectively) could be assigned, most of them in a sequential manner (100 and 118 correlations, respectively, in the inter‐residual hCAcoNH and hNcoCAH spectra were assigned for which the sequential walk sketched in Figure 1 B was performed successfully), the others by transferring the ^15^N and ^13^CA assignments obtained by ^13^C‐detected experiments to the ^1^H‐detected ones. The largest fraction of the NTD remains invisible in ^1^H‐detected spectra as well as in ^13^C detection.^44^

**Figure 1:**
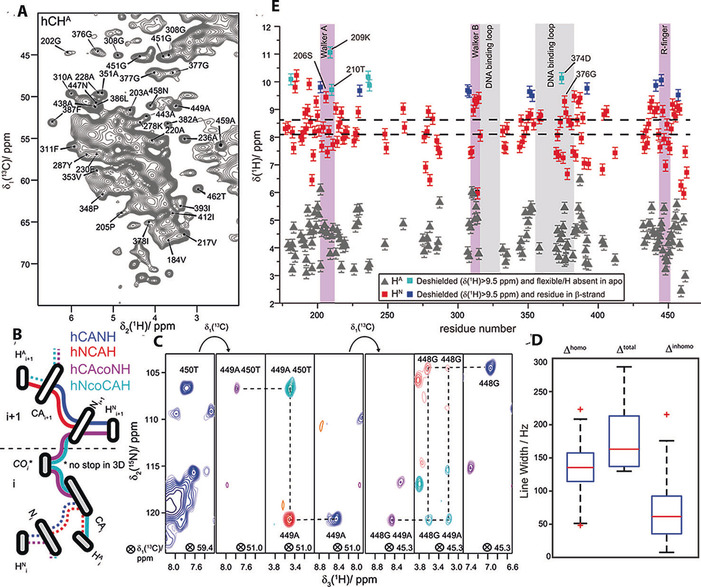
Sequential assignments of the proton resonances. A) 2D hCH spectrum with assignments of isolated resonances. B) Assignment strategy by using both, H^N^ and H^A^ protons. The dashed lines highlight connectivities at the same ^13^C resonance frequency. C) Representative 2D planes of 3D spectra used for sequential resonance assignment (backbone “walk”). D) Boxplots for the ^1^H homogeneous, the total, and the inhomogeneous line width, respectively (see also the Supporting Information). E) Site‐specific H^N^ and H^A^ chemical‐shift values. The dashed horizontal lines represent the average H^N^ shifts in α‐helices (*δ*=8.1 ppm) and β‐strands (*δ*=8.6 ppm).^47^ The error bars are estimated to 0.1 ppm.

**Figure 2:**
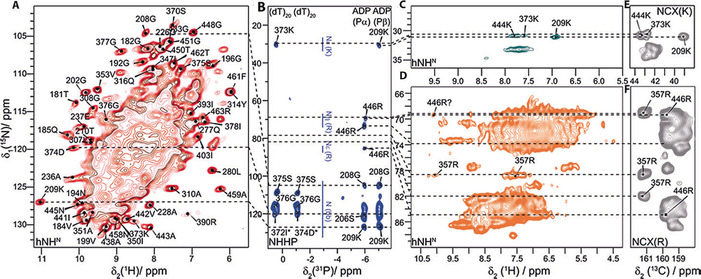
Solid‐state NMR spectra to probe protein–nucleotide interactions. A) 2D hNH spectrum with assignment of isolated resonances. B) NHHP spectrum with assigned resonances. The figure is taken in parts from reference 44. C) Lysine side chain 2D hNH spectrum with assignments. D) Arginine side chain 2D hNH spectrum with assignments. E) Lysine side chain 2D NC spectrum with assignments, from reference 44. F) Arginine side chain 2D NC spectrum with assignments, from reference 44. Dashed lines are guidance for the eye for the correlations discussed in the text.

The ease of ^1^H assignments strongly depends on the observed ^1^H line widths (Δ^tot^), which is the sum of homogeneous (Δ^homo^) and inhomogeneous (Δ^inhomo^) contributions^48^ (see the Supporting Information and Figures S2–S4). Figure 1 D shows the contributions to the ^1^H line widths determined for isolated peaks in the 2D hNH spectrum (see Figure S3). The average total line width of Δ^tot^=(200±50) Hz contains on average a homogeneous broadening of Δ^homo^=(140±40) Hz, which is comparable to other protonated systems.^48^, ^49^ The inhomogeneous contribution is on average Δ^inhomo^=(90±60) Hz. The assigned H^N^ and H^A^ chemical shifts are plotted in Figure 1 E, and are shown on a structural model of the CTD of the DnaB helicase^37^ in Figure 3, color coded with the corresponding ^1^H chemical‐shift values. Note that the H^N^ chemical shifts show a quite large spectral dispersion (≈5 ppm), which is attributed to the high sensitivity of proton shifts to noncovalent interactions: shielded resonances associated with ring‐current effects (for example, 314Y in the Walker B motif and 459A, 461F, and 463R possibly located in a loop above the ADP base plane) and deshielded resonances due to hydrogen‐bond formation (many of them located in β‐strands, see Figures 1 E, 3, and S5). Assignments of outlier resonances are shown in Figures 1 A and 2 A. For both ^1^H species residues in β‐strands were nearly completely assigned, whereas many residues in α‐helices remain unassigned. This is a consequence of the larger chemical‐shift dispersion of β‐strand residues, but also of the usually broader homogeneous ^1^H lines of residues in α‐helices due to a denser proton network.^15^

**Figure 3:**
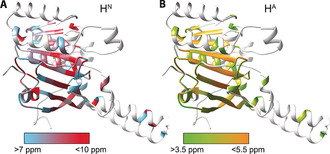
Proton assignment plotted on a structural model. A) Assigned H^N^ resonances plotted on a DnaB model based on the *Aa*DnaB:ADP complex (PDB ID: 4NMN) by using the color code shown in the legend. B) Assigned H^A^ resonances plotted on the same model by using the color code shown in the legend.

To further analyze nucleotide binding, spectra were recorded on two additional protein samples: the apo protein (no nucleotides bound) and DnaB:AMP‐PCP:DNA by using a pre‐hydrolytic ATP‐mimic.^44^

In order to complement the information from the chemical shift, we established direct polarization transfer between spins in spatial proximity (<8–9 Å), and performed a ^31^P‐detected 2D NHHP (Figure 2 B) correlation experiment (for a CHHP spectrum see Figure S6). Compared to the previously described ^31^P,^15^N TEDOR experiment used for identifying protein–RNA contacts in a smaller test system,^9^ the polarization in NHHP experiments is mediated between close‐by ^15^N and ^31^P nuclei through H–H spin diffusion, which allows to extract medium‐to‐long‐range structural restraints^50^ and is thus an alternative to TEDOR experiments. Sensitivity in such experiments is becoming an issue if the ^31^P spin concentration is small as in the investigated system. The spectra reveal correlations between phosphate groups and backbone amides, as well as arginine and lysine side chain nitrogen atoms. The spectrum clearly distinguishes different ^31^P shifts for ADP and DNA.^44^ As a matter of fact, assignments in the 2D spectrum remain ambiguous for such a large protein, but could be resolved considering the primary amino acid sequence and the motifs to which nucleotides were predicted to bind^37^ (i.e., residues 203A–210T, 445N–451G and residues 371D–382A,^51^ respectively), and taking also further NMR spectroscopic information into account (for example, CSPs and dynamic changes upon nucleotide binding, see Ref. 44).

The residue 209K is located in the Walker A motif. A clear 209K N^ζ^–ADP P^β^ cross signal can be observed in the NHHP spectrum (Figure 2 B), which positions the side chain to form a salt bridge to the β‐phosphate and/or to the AlF_4_^−^. And indeed, a side chain correlation peak involving ^15^N is observed for 209K in both the hNH and NC spectrum (Figures 2 C and E); observation of such cross signals is often related to involvement of the N^ζ^H_3_^+^ group in salt bridge formation.^52^ Interestingly, the 209K H^N^ chemical shift represents the most deshielded ^1^H resonance (*δ*=11.0 ppm) for the DnaB:ADP:AlF_4_^−^:DNA complex. Also, an (ambiguous) signal is observed at the 209K H^N^ shift in the NHHP spectrum. 209K H^N^ is thus with high confidence involved in a strong hydrogen bond, which is established only on nucleotide binding, because the 209K H^N^ resonance is not observed in the apo form. Such conclusion can be drawn from the hNH spectra shown in Figure 4, which compare the DnaB:ADP:AlF_4_^−^:DNA state with the pre‐hydrolytic DnaB:AMP‐PCP:DNA and the apo state. This agrees with previous findings that 209K stiffened upon ADP:AlF_4_^−^ binding and remained flexible in the apo form, interestingly also in the DnaB:AMP‐PCP:DNA state where one would also expect that 209K binds to the nucleotide (see Table S2 and Figure 4 for the 2D hNH spectra).^44^

**Figure 4:**
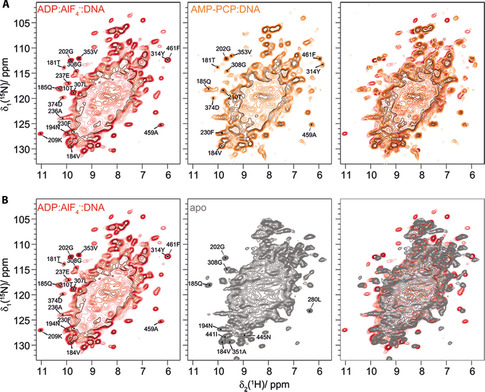
Deshielded and shielded ^1^H^N^ resonances differ between the DNA bound and apo state. A) Comparison of the 2D hNH spectra of DnaB:ADP:AlF_4_^−^: DNA (red) and DnaB:AMP‐PCP:DNA (orange). Characteristic deshielded and shielded isolated peaks discussed in the main text are marked. B) Comparison of the 2D hNH spectra of DnaB:ADP:AlF_4_^−^:DNA (red) and apo DnaB (gray). Characteristic peaks are marked.

The NHHP spectrum indicates spatial proximity of further 209K‐neighboring residues and the nucleotide among which 210T is most likely involved in hydrogen bonding (H^N^ shift of *δ*=9.7 ppm). 206S is also relatively deshielded (*δ*=9.4 ppm) for DnaB:ADP:AlF_4_^−^:DNA. The equivalent residue to 206S has been identified in other NTPases, whose structures locate this residue in the Walker A motif near the fluorine atom of the γ‐phosphate mimic, identifying its key role in stabilizing the γ phosphate during ATP hydrolysis.^53^

Besides 209K, correlation signals for N^η1/η2^ and N^*ϵ*^ side chain resonances are observed in the NHHP/CHHP spectra for 446R, showing that it is in close spatial proximity to Pα of ADP (Figures 2 B and S6). 446R is located in the R‐finger, which connects two adjacent monomers, and plays a central role in ATP binding and hydrolysis.^1^, ^53^, ^54^, ^55^ Although in the hNH spectrum (Figure 2 A) the 446R resonances cannot be unambiguously assigned, a deshielded ^1^H resonance (*δ*=10.1 ppm) is particularly observed for N^η1/η2^ at the corresponding ^15^N shift, which could support assignments to this residue based on the expected hydrogen‐bond formation with the ADP phosphate group.

The findings from the ^31^P intermolecular correlation experiments and ^1^H chemical shifts are summarized in Figure 5 A, and are compared to the interactions available from the *Bst*DnaB:GDP:AlF_4_^−^:DNA crystal structure (Figure 5 B). One can see that the contacts with the nucleotide as defined by NMR spectroscopy in DnaB are similar to those revealed by the X‐ray structure for *Bst*DnaB, as, for example, the close spatial proximity of the equivalent to 209K lysine side chain (216 K) located in the Walker A motif to the nucleotide. Despite these similarities, NMR spectroscopy also reveals small differences in the binding mode, as, for example, the spatial proximity of the R‐finger in DnaB:ADP:AlF_4_^−^:DNA (446R) exclusively to Pα as deduced from the NHHP and CHHP spectra (Figures 2 B and S6), which distinguishes it from *Bst*DnaB:GDP:AlF_4_^−^:DNA, where 420R is close to both GDP phosphate groups (Figure 5 B).^7^ Note that there is an important difference between the *Bst*DnaB structure and our NMR data for *Hp*DnaB regarding the occupancy of ATP binding sites. Whereas for *Bst*DnaB only five out of six nucleotide binding domains are occupied, full occupancy was derived from the NMR data for *Hp*DnaB^44^ still indicating structural differences between these proteins.

**Figure 5:**
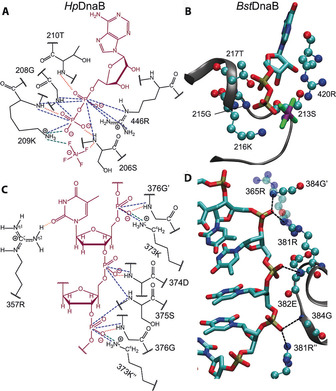
Protein–nucleotide interactions in DnaB derived from solid‐state NMR data. A) Schematic drawing of protein–ADP:AlF_4_^−^ (highlighted in magenta) contacts deduced from the NMR data (the dashed lines represent spatial correlations observed in the NMR spectra with the corresponding color code used for the spectra in Figure 2 and the dotted lines represent hydrogen bonds derived from chemical shifts). B) Protein–GDP:AlF_4_^−^ contacts in *Bst*DnaB:DNA as determined from the crystal structure (PDB ID: 4ESV^7^). C) Schematic drawing of protein–DNA contacts similar to A). ′ and ′′ indicate residues from the adjacent chains. D) Protein–DNA contacts in *Bst*DnaB:DNA as determined from the crystal structure (PDB ID: 4ESV^7^).

The DNA chemical‐shift region of the NHHP spectrum in Figure 2 B reveals two ^31^P chemical shifts for two ^31^P DNA phosphate resonances. The binding of two DNA nucleotides per DnaB monomer is a common feature of SF4 helicases^7^, ^44^ and reflected in solid‐state NMR spectra by two ^31^P resonances with different ^31^P chemical‐shift values (see Figure 6 for a 2D 150 ms ^31^P–^31^P DARR spectrum). Strong crosspeaks between the two ADP resonances indicate the close spatial proximity of the two ADP phosphate groups, whereas for the two DNA resonances such crosspeaks are less intense. The presence of the crosspeaks however still indicates that the two ^31^P DNA resonances can be assigned to two structurally distinct but neighbored phosphate groups of DNA.

**Figure 6:**
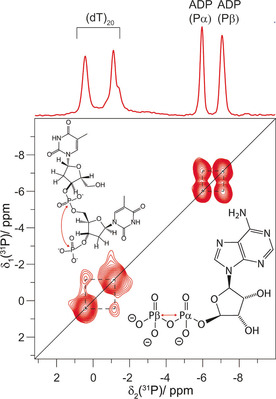
Two DNA nucleotides bind per DnaB monomer and are in spatial proximity. ^31^P–^31^P 150 ms DARR spectrum of DnaB:ADP:AlF_4_:DNA showing that the two bound DNA nucleotides are in close spatial proximity. On top of the 2D spectrum, a ^31^P CP spectrum is shown (taken from ref. 44).

Only one shows a correlation between the ^31^P DNA resonance at highest ppm values and the lysine side chain of 373K (see Figure S7 for the assignment of this residue). The N^ξ^−H^ξ^ side chain resonance of this residue is also observed in the hNH spectrum (*δ*=7.6 ppm, Figure 2 C) supporting its involvement in a salt bridge with the DNA phosphate. For apo DnaB, no lysine side chain correlations are detected, in line with our previous findings.^44^ Concomitant to this, the neighboring residue, 374D shows a particularly deshielded resonance of *δ*=10.1 ppm, indicating that it is as well involved in a hydrogen bond, possibly with the DNA (Figures 4 and 5, Table S2).

Further interactions can be identified in the arginine side chain region of the hNH spectrum in Figure 2 D. The isolated 357R ^15^Nη2 chemical shift (Figures 2 F and S8) can be clearly identified. Two correlation peaks to ^1^H are detected at this frequency, one around *δ*=10.2 ppm, and the other around *δ*=7.6 ppm (Figure S9). Whereas the latter is a typical amide proton chemical‐shift value, the first is deshielded, indicating hydrogen‐bonding interactions, but no correlations to the DNA phosphates are observed in NHHP (Figure 2 B) and only very weak ones are seen in the CHHP spectra, in contrast to 446R of the R‐finger (Figure S6). Arginine residues can show three different side chain–DNA binding modes:^4^, ^6^ either to the DNA phosphate group, to the base edges, or to the ribose, through hydrogen bonding or electrostatic interactions or to the DNA base plane by electrostatic cation–π interactions.^56^, ^57^, ^58^ The absence of NHHP correlations, combined with the deshielded proton shift, positions 357R in coordination with the DNA base edge. As only one H^N^ is deshielded, only one N^η^H_2_ moiety is involved in a hydrogen bond to one of the thymidine oxygen atoms (see Figure 5 C for a schematic drawing). The relatively broad resonances (predominantly in the ^1^H dimension) might be a consequence of structural disorder, for example, a small structural inequivalence of the DnaB monomers in the oligomeric protein‐DNA complex, leading to a chemical‐shift distribution. These observations show that NMR spectroscopy allows us to distinguish between hydrogen bonds to the DNA phosphate groups or to the DNA base edge. Arginine side chain–thymidine DNA base edge interactions are rather rarely observed and much less frequent than, for example, arginine–guanidine pairs.^4^, ^6^

Figure 5 C schematically summarizes the obtained information on the DNA binding mode of DnaB, and compares it to the DNA binding modes from equivalent residues in the *Bst*DnaB:GDP:AlF_4_^−^:DNA structure.^7^ 373K contacts the DNA phosphate backbone through a salt bridge, with nearby residues 374D–376G among which 374D and 376G are potentially also involved in binding based on their deshielded H^N^ resonances. 357R contacts possibly the DNA base edge through hydrogen bonding. Figure 5 D shows that equivalent contacts can be seen in the crystal structure of *Bst*DnaB:GDP:AlF_4_^−^:DNA^7^), where the side chain of 381R (corresponds to 373K in *Hp*DnaB) is in spatial proximity to the phosphate group of DNA, whereas the amide nitrogen atoms of 382E and 384G (374D and 376G, respectively, in *Hp*DnaB) contact the phosphate group of DNA possibly through hydrogen bonds. However, the analogue of 357R in *Bst*DnaB (365R) is close to the DNA phosphate groups in the crystal structure indicating still differences in DNA coordination.

In conclusion, we illustrated that a large part of the ^1^H resonances in a motor protein assembly can be assigned by exploiting the well‐dispersed H^N^ and H^A^ frequencies in a combined 3D assignment approach by using four different spectra. We showed that NHHP spectra can be recorded with sufficient signal/noise, even if acquisition times remain long and demonstrated how information from both can be combined to identify and conclude on nucleotide binding modes, for both ATP and DNA. Our findings compare well with data on a related protein whose crystal structure is available, and validates the presented approach. The procedure described here shall thus allow to detect noncovalent interactions in molecular recognition processes involving nucleotides also in further noncrystalline protein assemblies, be it in the context of nucleic acid synthesis, extension, repair, or packaging, as typically occurring by capsids in viruses or with histones in chromosomes. Even the investigation of changes in protein–DNA contacts during functional cycles, for example, in DNA replication, becomes accessible.

## Conflict of interest

*The authors declare no conflict of interest*.

## Supporting information

As a service to our authors and readers, this journal provides supporting information supplied by the authors. Such materials are peer reviewed and may be re‐organized for online delivery, but are not copy‐edited or typeset. Technical support issues arising from supporting information (other than missing files) should be addressed to the authors.

Supplementary
