# Supplementary material for: Nucleotide Binding Modes in a Motor Protein Revealed by 31P‐ and 1H‐Detected MAS Solid‐State NMR Spectroscopy
Source: Chembiochem. 2019 Sep 30;21(3):324–30. doi: 10.1002/cbic.201900439 (PMC7318265; doi:10.1002/cbic.201900439)
Supplement: Supplementary file 1 — Supplementary [file CBIC-21-324-s001.pdf]

CHEMBIOCHEM

Supporting Information

Nucleotide Binding Modes in a Motor Protein Revealed by ^{31}P- and ^{1}H-Detected MAS Solid-State NMR Spectroscopy

Thomas Wiegand,*[a] Maarten Schledorn,[a] Alexander A. Malär,[a] Riccardo Cadalbert,[a] Alexander Däpp,[a] Laurent Terradot,[a] Beat H. Meier,[a] and Anja Böckmann*[b]

cbic_201900439_sm_miscellaneous_information.pdf

Materials and Methods

Sample preparation

AMP-PCP and ADP was purchased from Sigma-Aldrich and $(dT)_{20}$ from Microsynth.

Expression and purification of HpDnaB

The protein was cloned into the vector pACYC-duet1 (using the forward primer 5'-agtcatatggatcatttaaagcatttgcag-3' containing a *Nde*I restriction site and reverse primer 5'-atactcgagttcaagttgtaactatatcataatcc-3' containing a *Xho*I site)[1], and expressed in the *E. coli* strain BL21 Star (DE3) (One Shot® BL21 Star™ (DE3) Chemically Competent *E. coli*, Invitrogen™). The overexpression was performed in M9 minimal medium[2] using ^{13}C-enriched glucose 2 g.L^{-1} (Cambridge Isotope Laboratories, Inc. CLM-1396-PK) and ^{15}N-enriched ammonium chloride 2 g.L^{-1} (Sigma-Aldrich® 299251) as sole carbon and nitrogen sources. The cells were lysed by a microfluidization process. ^{13}C-^{15}N labelled *Hp*DnaB was purified by heparin-agarose affinity chromatography using a 5 mL HiTrap Heparin HP column (GE Healthcare Life Sciences) followed by anion exchange chromatography using a 5 mL HiTrap Q HP column (GE Healthcare Life Sciences). The purified protein was concentrated up to 30 mg.mL^{-1} by centrifugation in buffer A (2.5 mM sodium phosphate, pH 7.5, 130 mM NaCl). For more details see reference [3].

Preparation of HpDnaB:nucleotide:DNA complexes

The *Hp*DnaB:nucleotide complexes were prepared by incubating DnaB with 5 mM $MgCl_2$ * $6H_2O$ and 5 mM of nucleotide for 2 h at 4°C. In the case of ADP:AlF_4^-, an NH_4AlF_4 solution was prepared by incubating 1 M $AlCl_3$ solution with a 6-fold excess of 1M NH_4F solution (compared to $AlCl_3$) for 5 min. in H_2O. 1 mM of $(dT)_{20}$ was added to the complexes and reacted for 30 min at r.t.. The protein solution was sedimented in the MAS-NMR rotor (16 h at 4°C at 210'000 g).

Solid-state NMR experiments

^{1}H-detected solid-state NMR spectra were acquired at 20.0 T static magnetic field strength using a 0.7 mm Bruker probe. ^{13}C-detected spectra were recorded at the same magnetic field, but in a 3.2 mm Bruker "Efree" probe[4]. The MAS frequency was set to 105.0-110.0 kHz. The 2D and 3D spectra were processed with the software TOPSPIN (version 3.5, Bruker Biospin) with a shifted (2.5 to 3.5) squared cosine apodization function and automated baseline correction in the indirect and direct dimensions. ^{1}H-detected spectra were cut in the F1-

dimension after 1k points. ^{31}P-detected experiments were acquired at 11.74 T in a Bruker 3.2 mm probe (using home-build $^{31}P/^{13}C$ and $^{31}P/^{15}N$ inserts) using a spinning frequency of 17.0 kHz. ^{31}P-detected experiments were processed with an exponential line broadening of 10 Hz in the two dimensions. The spectra were referenced to 85% H_3PO_4. The sample temperature was set to 278 K[5]. All spectra were analysed with the software CcpNmr[6-8] and referenced to 4,4-dimethyl-4-silapentane-1-sulfonic acid (DSS). All experimental details are provided in Table S3.

^{1}H line-widths of DnaB are dominated by homogeneous broadening effects

The ^{1}H line-width in solid-state NMR spectra can be split up in homogeneous and inhomogeneous contributions[9-12]. Homogeneous line broadening can be coherent (induced by H-H homonuclear dipolar interactions) or incoherent (induced by molecular motions)[11, 13]. This results in the following equation: $\Delta^{total}=\Delta^{homo}+\Delta^{inhomo}= \Delta^{coherent}+\Delta^{incoherent} +\Delta^{inhomo}$. Inhomogeneous effects are related to sample inhomogeneities such as disorder and crystal defects leading to chemical-shift distributions, or B_0 inhomogeneities and contributions from an imperfect probe shimming[11-12]. While homogeneous interactions scale down with the MAS frequency[11-12, 14], the inhomogeneous contributions remain uninfluenced[11]. Experimentally, homogeneous broadening effects are accessible by spin-echo decay curves[15], revealing the T_2' relaxation times which are related by $\Delta^{homo} = 1/(\pi T_2')$ and $\Delta^{total} = \Delta^{homo} + \Delta^{inhomo}$ to the total NMR line width at half maximum (FWHM). Figure S2 shows the site-specific homogeneous contributions to the H^N line-widths determined from 2D spin-echo experiments (blue bars, only isolated resonances were analyzed). The homogeneous contribution for the 52 isolated peaks has an average value of 140±40 Hz (Figure 1d). This value is similar to the homogeneous line-widths observed for other fully protonated systems such as ubiquitin (96±6 Hz at 126 kHz MAS)[11], GB1 (120±30 Hz at 111 kHz MAS)[16] and AP205 nucleocapsids (140±40 Hz at 111 kHz MAS)[16]. Faster spinning would thus clearly result in a further reduction of ^{1}H NMR line-widths[11-12]. Figure S2 shows as well the total line width for a set of 20 isolated peaks in the 2D hNH spectrum (orange bars), which varies around a mean value of 200±50 Hz (Figure 1d). For the spectrum and assignment used for the analysis, as well as the set of isolated peaks, refer to Figure S3. The inhomogeneous contribution for these 20 peaks is obtained by subtracting the homogeneous contribution from the total NMR line-width and has an average of 90±60 Hz (Figure 1d). Therefore, an inhomogeneous contribution to the line-widths exists for DnaB which is expected to remain uninfluenced by faster MAS spinning frequencies[11]. This is distinct to crystalline ubiquitin for which the inhomogeneous contribution is in the order of only a few Hz[11], but similar to GB1[16] or SH3[17] for which inhomogeneous contributions up to 70 Hz were reported. We attribute the inhomogeneous broadening observed for DnaB to imperfect probe shimming, the symmetry of the hexamer (C3) inducing an (unresolved) peak splitting and conformational inhomogeneities in the sediment. To which extent the latter explanation influences the NMR line-widths still has to be determined, but the strongly varying residue-specific values might imply that the last two factors are more important, since the first (probe shimming) would lead to a constant offset in the line-widths. Note however, that the determination of the inhomogeneous contribution is also slightly influenced by the signal

processing. For our analysis we used a quadratic shifted sine bell apodization function (QSINE) with an SSB of 2.5, which improves the spectral quality but still gives similar line width results compared to the case where no window function is applied (for more details on how processing changes the extracted total line width of the herein studied system see Figure S4).

Supplementary Figures

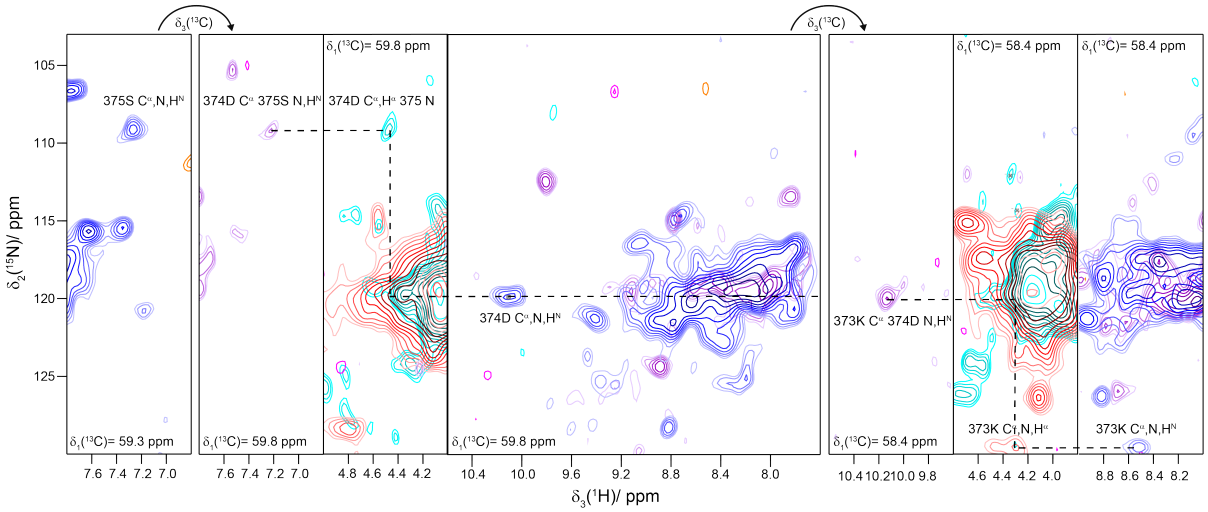

Figure S1: *Assignment ambiguities in ^{1}H-detected spectra*. Example for a sequential walk along residues 375S-373K using the assignment scheme presented in Figure 1b. Assignment ambiguities are resolved by using additional the information from ^{13}C-detected assignment experiments[18].

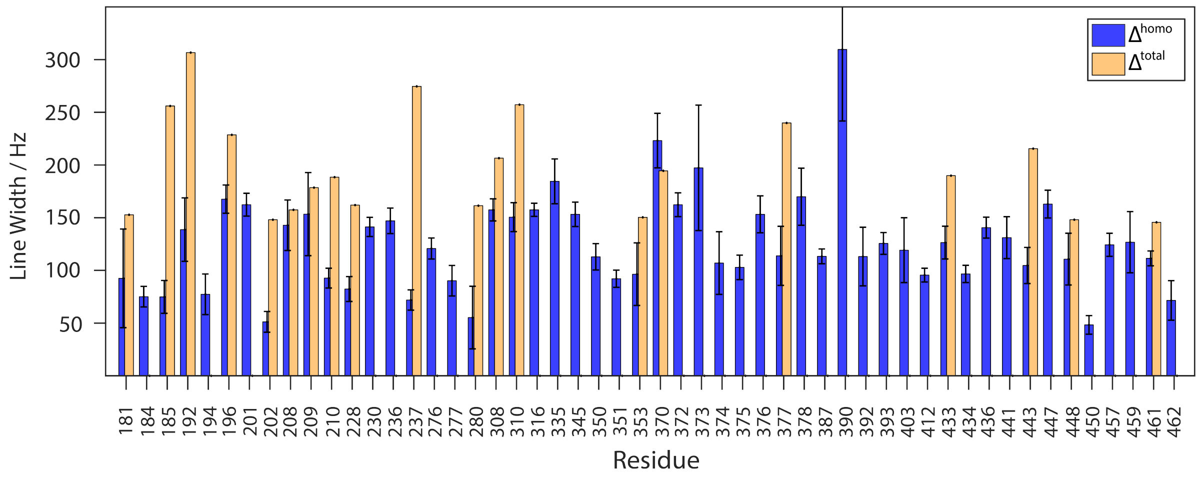

Figure S2: *^{1}H line-widths in solid-state NMR spectra of DnaB are dominated by homogeneous contributions.* Comparison of site-specific homogeneous contributions (blue bars) vs. total experimental linewidths for a set of isolated resonances (orange bars).

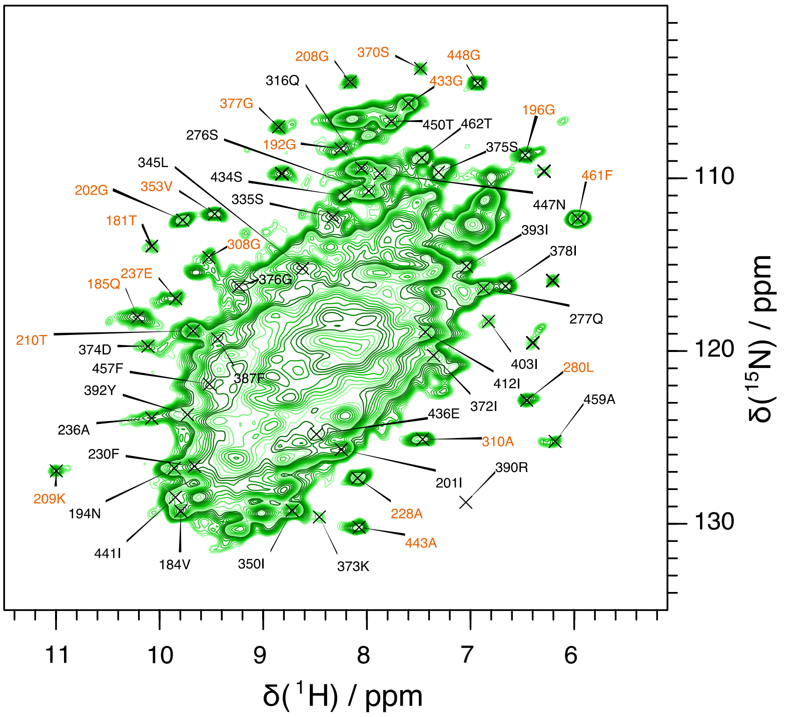

Figure S3: Reference 2D hNH spectrum and assignment used for the T_2' analysis. Orange peaks indicate those peaks for which a total line width could be extracted.

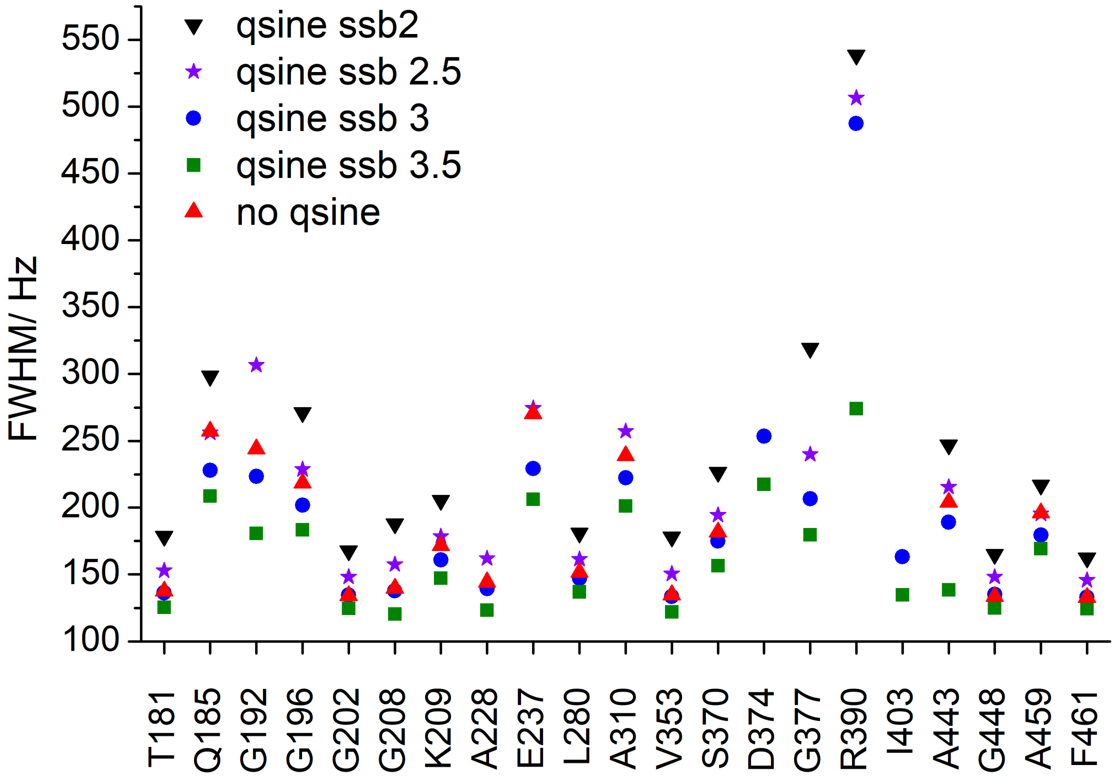

Figure S4: *Signal processing influences ^{1}H line-widths extracted from 2D spectra.* ^{1}H FWHM for a selection of isolated peaks taken from the hNH spectrum shown in Figure 2a. For the determination of the inhomogeneous contribution to the line-width a qsine window function (ssb 2.5) was used which is for most residues very close to the value without window function (no qsine). Note that some residues have a larger (nonphysical) FWHM than 575 Hz most likely due to peak overlap and are thus not shown here. The spectrum in Figure 2a is processed with a qsine (ssb 3) window function.

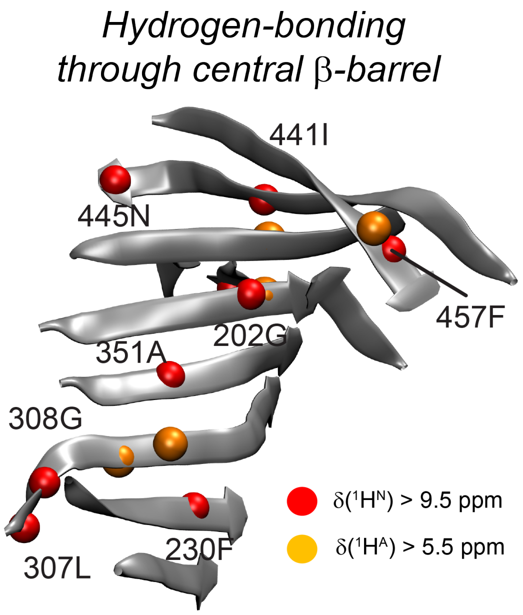

Figure S5: *Deshielded HN and HA resonances form a band through the central β-barrel.* Residues with deshielded ^{1}H resonances (see legend) plotted on the central half-β barrel (taken from a DnaB model[1]). The residues form a network through this structural element.

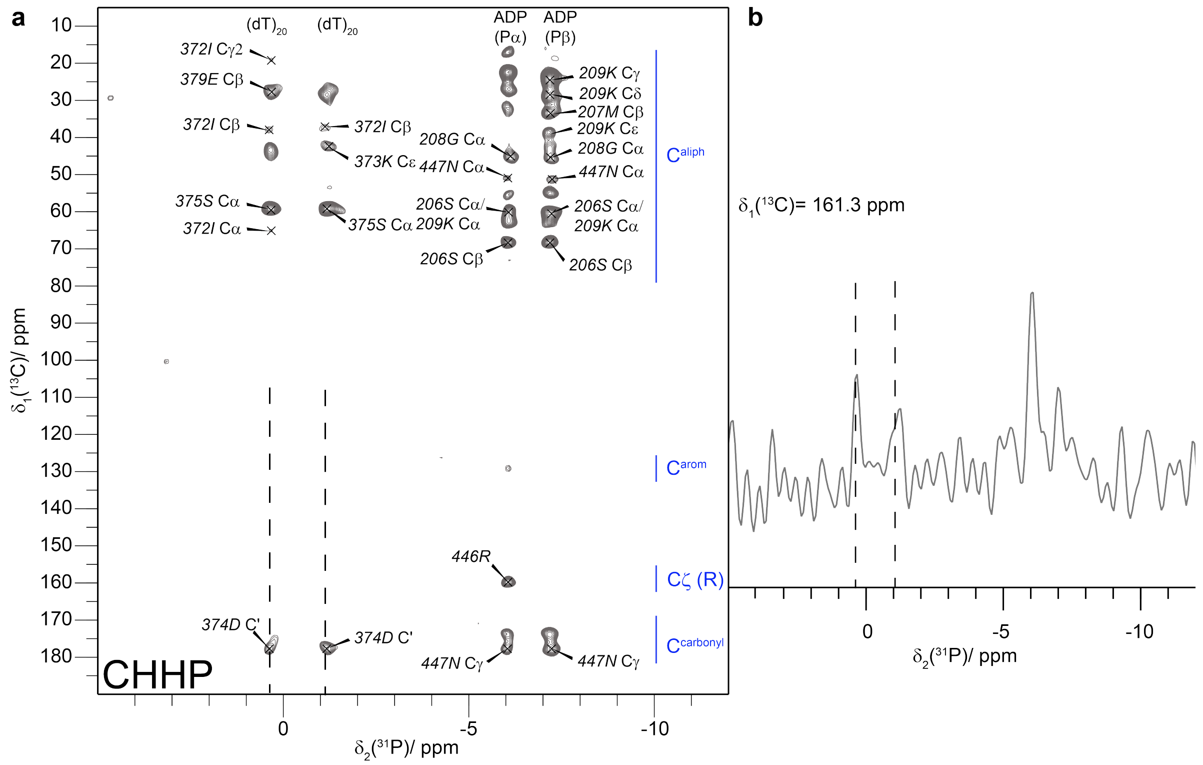

Figure S6: *CHHP to probe protein-nucleotide contacts.* **a** CHHP spectrum of DnaB:ADP:AlF_4^-:DNA with resonance assignments. **b** Slice along F2 of the CHHP 2D spectrum at δ(^{13}C)= 161.3 ppm (357R Cζ value). A very weak DNA-arginine correlation appears which is significantly weaker than the one observed for 446R indicating that 357R is not in very close spatial proximity to the DNA phosphate groups. The dashed lines highlight the DNA ^{31}P resonances.

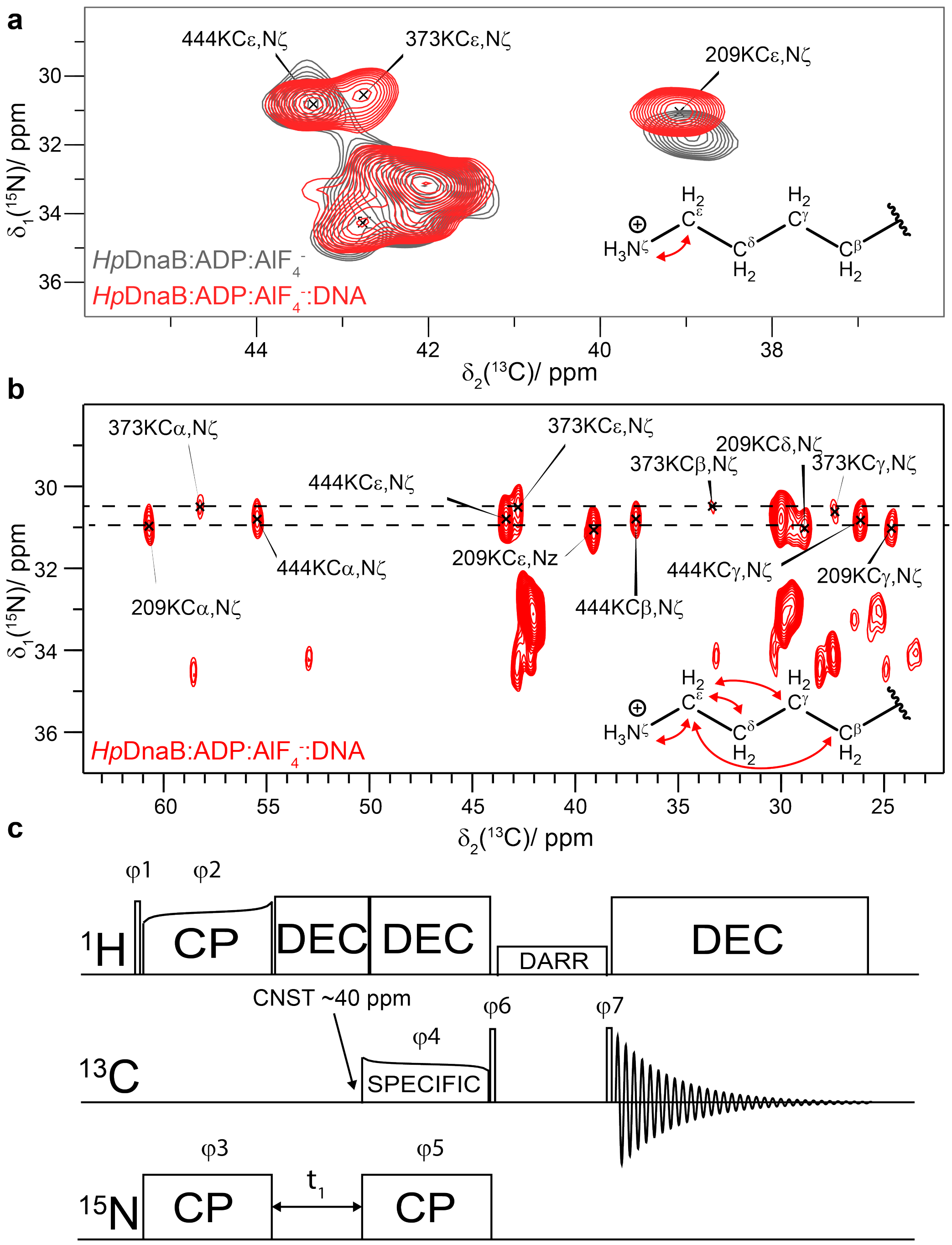

Figure S7: *Lysine 373K becomes rigid upon DNA binding and coordinates to the DNA.* **a** ^{15}N,^{13}C correlation spectra for the lysine sidechain region of DnaB:ADP:AlF_4^- and DnaB:ADP:AlF_4^-:DNA (the latter spectrum is from reference [18]). **b** N-CC correlation spectrum (for the pulse sequence see label **c**) allowing to unambiguously assign 373K. The following phases were used: φ1= y –y, φ2= x, φ3= x, φ4=x x x x –x –x –x –x y y y y –y –y –y –y, φ5= x x –x –x, φ6= y y y y –y –y –y –y –x –x –x –x x x x x, φ7= -y –y –y –y y y y y x x x x –x –x –x –x, $φ_{rec}$= x –x –x x –x x x –x y –y –y y –y y y –y. The dashed lines represent the ^{15}Nζ chemical-shift values of 209K and 373K.

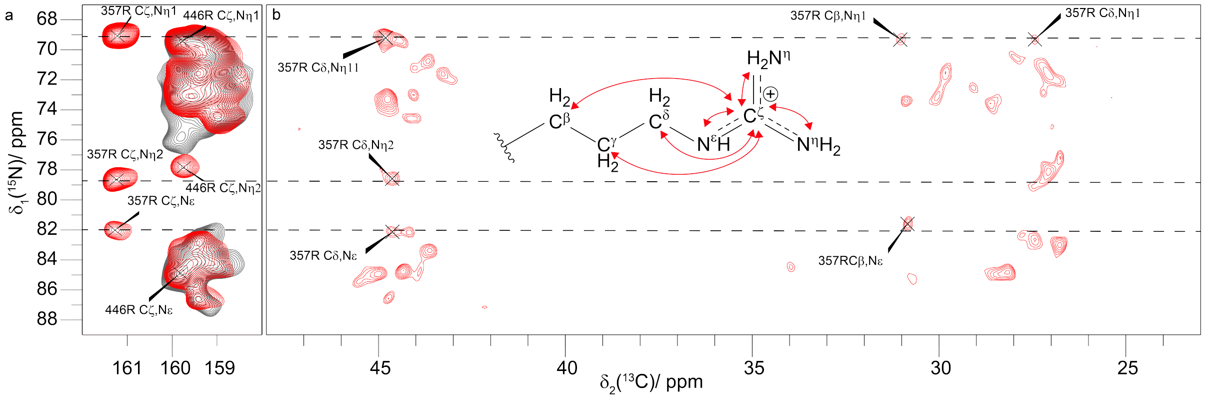

Figure S8: *Arginine 357R becomes rigid upon DNA binding and is not located in the NBD.* **a** ^{15}N,^{13}C correlation spectra for the arginine sidechain region of DnaB:ADP:AlF_4^- (shown in gray) and DnaB:ADP:AlF_4^-:DNA (red). The red spectrum is taken from reference[18]. **b** N-CC correlation spectrum (for the pulse sequence see Figure S7 with the SPECIFIC CP step optimized for the arginine sidechain resonances) allowing to unambiguously assign 357R.

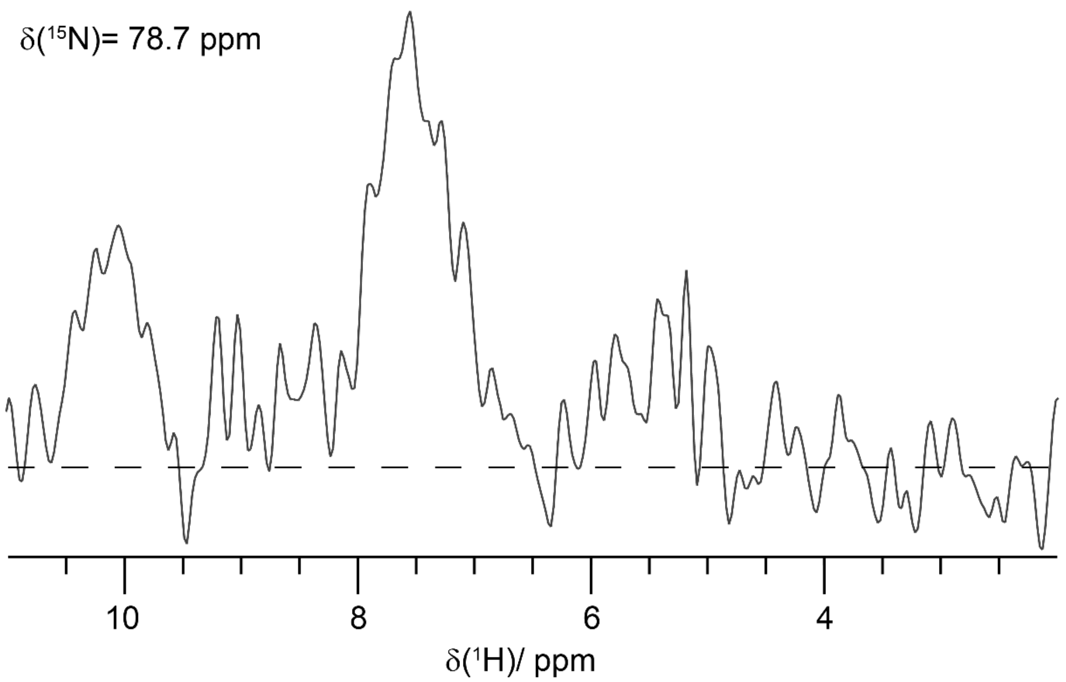

Figure S9: *Arginine 357R is involved in hydrogen bonding via its sidechain.* 1D trace along F2 (δ(^{15}N)= 78.7 ppm) of a 2D (H)NH spectrum of DnaB:ADP:AlF4-:DNA recorded at 110 kHz.

Table S1: *Summary of H^N and H^A chemical-shift values of DnaB:ADP:ALF$_4^-$:DNA.* The values are graphically shown in Figure 1e.

Residue	amino acid	δ(H^N) ± 0.1/ ppm	δ(H^A) ± 0.1/ ppm
177	Thr	8.3	-
179	Ile	8.4	4.1
180	Pro	-	-
181	Thr	10.1	4.0
182	Gly	8.2	3.2, 4.1
183	Phe	7.6	4.7
184	Val	9.8	3.7
185	Gln	10.2	-
188	Asn	8.0	4.4
189	Tyr	7.9	4.3
190	Thr	8.2	4.3
191	Ser	8.9	4.1
192	Gly	8.3	3.1, 3.6
193	Phe	9.0	5.4
194	Asn	9.9	4.8
195	Lys	8.6	4.5
196	Gly	6.5	3.2, 4.2
197	Ser	7.3	4.6
198	Leu	8.2	5.3
199	Val	9.3	4.8
200	Ile	8.8	5.5
201	Ile	8.2	5.2
202	Gly	9.8	6.1, 3.3
203	Ala	7.7	4.6
204	Arg	8.3	-
205	Pro	-	4.8
206	Ser	9.4	4.6
207	Met	7.5	4.5
208	Gly	8.2	-
209	Lys	11.0	3.8
210	Thr	9.7	4.4
211	Ser	7.7	4.7
213	Met	9.0	3.8
214	Met	7.8	4.6
215	Asn	8.4	4.5
216	Met	8.5	4.3
217	Val	8.9	3.3
218	Leu	7.9	4.0
219	Ser	8.0	4.2
220	Ala	8.2	4.1
221	Leu	8.2	-
226	Gly	7.9	3.2

227	Val	8.1	4.8
228	Ala	8.1	5.4
229	Val	8.9	4.9
230	Phe	9.7	5.4
236	Ala	10.2	3.3
237	Glu	9.9	3.4
248	Thr	8.1	4.1
249	Ser	7.6	-
261	Asp	9.0	4.9
262	Asp	8.4	-
275	Leu	-	4.3
276	Ser	8.0	3.9
277	Gln	6.9	-
278	Lys	8.2	4.4
279	Lys	8.9	4.1
280	Leu	6.5	4.8
282	Phe	8.7	-
285	Lys	7.9	4.0
286	Ser	7.6	4.2
287	Tyr	7.4	5.6
307	Leu	9.7	3.8
308	Gly	9.6	3.7, 4.9
309	Ile	7.4	4.7
310	Ala	7.5	6.0
311	Phe	9.0	5.9
312	Ile	9.3	5.0
313	Asp	9.2	5.5
314	Tyr	6.0	5.1
315	Leu	9.4	3.8
316	Gln	8.0	-
332	Ala	8.2	3.9
333	Glu	7.6	-
334	Ile	7.6	3.5
335	Ser	8.3	3.8
344	Glu	7.6	4.0
345	Leu	8.6	4.2
346	Glu	7.8	3.8
347	Ile	8.0	-
348	Pro	-	5.3
349	Ile	8.3	4.4
350	Ile	8.7	4.9
351	Ala	9.6	5.3
352	Leu	8.8	5.5
353	Val	9.5	5.4
354	Gln	8.5	-
358	Ser	8.7	4.0

359	Leu	8.8	-
370	Ser	7.4	4.0
371	Asp	8.1	4.6
372	Ile	7.3	3.7
373	Lys	8.5	4.3
374	Asp	10.1	4.5
375	Ser	7.2	4.8
376	Gly	9.3	3.5, 5.4
377	Gly	8.9	3.6, 4.0
378	Ile	6.7	4.1
379	Glu	8.6	-
381	Asp	8.5	-
382	Ala	7.5	3.7
383	Asp	9.5	4.5
384	Ile	7.3	4.7
385	Val	8.6	4.6
386	Leu	9.3	5.4
387	Phe	9.4	6.5
388	Leu	8.0	5.1
389	Tyr	8.7	4.8
390	Arg	7.1	4.7
391	Gly	-	3.4, 3.9
392	Tyr	9.8	4.3
393	Ile	7.0	3.4
403	Ile	6.8	4.2
404	Asp	8.8	4.1
406	Leu	8.9	-
412	Ile	7.4	3.6
432	Asn	8.0	4.8
433	Gly	7.6	4.2
434	Ser	8.2	-
435	Ile	7.8	4.4
436	Glu	8.5	4.8
437	Glu	9.1	4.8
438	Ala	9.3	5.5
439	Glu	-	4.6
440	Ile	8.9	4.5
441	Ile	9.9	4.0
442	Val	9.0	4.1, 4.5
443	Ala	8.1	4.2
444	Lys	7.6	4.4
445	Asn	10.1	4.7, 4.9
446	Arg	8.9	3.9
447	Asn	7.9	5.4
448	Gly	7.0	3.2, 3.6
449	Ala	8.5	3.5

450	Thr	7.8	4.1
451	Gly	7.6	3.8, 4.4
452	Thr	8.3	4.5
453	Val	9.2	4.2
454	Tyr	8.8	4.8
455	Thr	8.8	5.1
456	Arg	9.1	5.6
457	Phe	9.5	4.7
458	Asn	9.0	4.1
459	Ala	6.3	2.4
461	Phe	6.0	5.0
462	Thr	7.5	3.0
463	Arg	6.7	-

Table S2: *Summary of deshielded $^1H^N$ resonances.* Residues with $^1H^N$ chemical-shift values > 9.3 ppm are given. Residues highlighted in green are part of the central beta-barrel. Resonances classified as flexible are not visible in the spectra, most likely due to dynamics. Resonances classified as H absent are visible in ^{13}C-detected spectra, but remain unassigned in the ^1H dimension.

Residue	$\delta(^1H^N) \pm 0.1$/ ppm DnaB:ADP:AlF_4^-:DNA	$\delta(^1H^N) \pm 0.1$/ ppm DnaB:AMP-PCP:DNA	$\delta(^1H^N) \pm 0.1$/ ppm apo DnaB
209K	11.0	flexible	flexible
185Q	10.2	10.4	10.4
236A	10.2	H absent	flexible
374D	10.1	10.2	flexible
181T	10.1	10.0	flexible
445N	10.1	9.5	9.0
194N	9.9	9.9	9.8
237E	9.9	9.7	H absent
441I	9.9	9.8	9.8
184V	9.8	9.9	9.8
202G	9.8	9.7	9.7
392Y	9.8	9.8	9.0
210T	9.7	9.8	H absent
307L	9.7	9.7	flexible
230F	9.7	9.8	flexible
351A	9.6	9.6	9.6
308G	9.6	9.6	9.7
457F	9.5	9.5	9.0
353V	9.5	9.4	H absent
383D	9.5	9.2	7.6
206S	9.4	flexible	flexible

Table S3: *Experimental solid-state NMR parameters.*

**Experiment**	**NHHP**	**CHHP**
MAS frequency/ kHz	17	17
Field/ T	11.7	11.7
Transfer I	HN-CP	HC-CP
^{1}H field/ kHz	45	60
X field/ kHz	29.6	42.0
Shape	Tangent ^{1}H	Tangent ^{1}H
Time/ ms	0.4	0.5
Transfer II	NH-CP	CH-CP
^{1}H field/ kHz	45	60
^{15}N field/ kHz	29.6	42.0
Shape	Tangent ^{1}H	Tangent ^{1}H
Time/ ms	0.4	0.5
Transfer III	H-H SD	H-H SD
Time/ms	0.2	0.2
Transfer IV	HP-CP	HP-CP
^{1}H field/ kHz	60	60
^{31}P field/ kHz	44.7	41.2
Shape	Tangent ^{1}H	Tangent ^{1}H
Time/ ms	1.5	1.2
t2 increments	3072	3072
Sweep width (t2)/ kHz	125	125
Acquisition time (t2)/ ms	12.3	12.3
t1 increments	56	96
Sweep width (t1)/ kHz	7.1	25
Acquisition time (t1)/ ms	3.9	1.9
^{1}H Spinal64 decoupling power/ kHz	90	90
Interscan delay/ s	2	2
Number of scans	9984	5632
Measurement time/ h	311	300

**Experiment**	**NCX (K) (ADP:A lF_4^- :DNA)**	**NCX (K) (ADP:A lF_4^-)**	**NCC (K) (ADP:A lF_4^- :DNA)**	**NCX (R) (ADP:A lF_4^-)**	**NCX (R) (ADP:A lF_4^- :DNA)**	**NCC (R) (ADP:A lF_4^- :DNA)**
MAS frequency/ kHz	17	17	17	17	17	17
Field/ T	20	20	20	20	20	20
Transfer I	HN-CP	HN-CP	HN-CP	HN-CP	HN-CP	HN-CP
^{1}H field/ kHz	60	60	60	60	60	60
X field/ kHz	42.4	43.6	43.4	40	40	43.4
Shape	Tangent ^{1}H	Tangent ^{1}H	Tangent ^{1}H	Tangent ^{1}H	Tangent ^{1}H	Tangent ^{1}H
Time/ ms	1.2	1.6	1.4	0.7	0.7	0.7
Transfer II	NC-CP	NC-CP	NC-CP	NC-CP	NC-CP	NC-CP
^{13}C field/ kHz	6	6	6	6	6	6
^{15}N field/ kHz	20.4	21.5	21.5	9.4	9.4	20.7
Shape	Tangent ^{13}C	Tangent ^{13}C	Tangent ^{13}C	Tangent ^{13}C	Tangent ^{13}C	Tangent ^{13}C
Carrier/ ppm	42	42	42	159.3	159.3	159.3
Time/ ms	7	7	7	3	3	3.5
Transfer III	-	-	DARR	-	-	DARR
^{1}H field/kHz	-	-	17.0	-	-	17.0
Tims/ ms	-	-	125	-	-	100
t1 increments	1792	1792	1792	1536	1536	1536
Sweep width (t1)/ kHz	66.7	66.7	66.7	66.7	66.7	66.7
Acquisition time (t1)/ ms	13.4	13.4	13.4	11.5	11.5	11.5
T2 increments	3072	3072	3072	3072	3072	3072
Sweep width (t2)/ kHz	100	100	100	100	100	100
Acquisition time (t2)/ ms	15.4	15.4	15.4	15.4	15.4	15.4
^{1}H Spinal64 decoupling power/ kHz	90	90	90	90	90	90
Interscan delay/ s	2.7	2.7	3	2.5	2.5	3
Number of scans	16	16	32	24	16	32
Measurement time/ h	22	22	48	27	18	41

**Experiment**	**hNH (apo)**	**hNH (AMP-PCP:DNA)**	**hNH (ADP:AlF4-:DNA)**	**hCH (ADP:AlF4-:DNA)**
MAS frequency/ kHz	108	105	110	110
Field/ T	20	20	20	20
Transfer I	HN-CP	HN-CP	HN-CP	HC-CP
^{1}H field/ kHz	135	135.0	85	89
X field/ kHz	36	34.6	15	15
Shape	Tangent ^{1}H	Tangent ^{1}H	Tangent ^{1}H	Tangent ^{1}H
^{15}N carrier/ ppm	117.5	117.5	78	56
Time/ ms	0.6	0.6	1.0	0.9
Transfer II	NH-CP	NH-CP	NH-CP	CH-CP
^{1}H field/ kHz	135	135.0	85	89
^{15}N field/ kHz	36	34.6	-	15
^{13}C field/ kHz	-	-	15	-
Shape	Tangent ^{1}H	Tangent ^{1}H	Tangent ^{1}H	Tangent ^{1}H
1H carrier/ ppm	4.8	4.8	4.8	4.8
Time/ ms	1.0	1.5	1.0	0.7
t1 increments	620	512	512	488
Sweep width (t1)/ ppm	180	180	180	190
Acquisition time (t1)/ ms	20.0	16.5	16.5	6.0
t2 increments	1024	5550	1024	1024
Sweep width (t2)/ ppm	46.7	43.2	40.0	46.7
Acquisition time (t2)/ ms	12.9	69.9	15.0	12.9
^{1}H swfTPPM decoupling/ kHz	10	10	10	10
^{15}N WALTZ64 decoupling/ kHz	5.0	5.0	5.0	-
^{13}C WALTZ64 decoupling/ kHz	-	-	-	5.0
Interscan delay/ s	1.2	1.0	1.2	0.9
Number of scans	64	184	658	120
Measurement time/ h	15	27	128	18

Experiment	(ADP:AlF$_4^-$:DNA)			
	**hCANH**	**hCAcoNH**	**hNCAH**	**hNcoCAH**
MAS frequency/ kHz	110	110	110	110
Field/ T	20	20	20	20
Transfer I	HC-CP	HC-CP	HN-CP	HN-CP
^{1}H field/ kHz	89	89	85	85
X field/ kHz	15	15	15	15
Shape	Tangent ^{1}H	Tangent ^{1}H	Tangent ^{1}H	Tangent ^{1}H
^{15}N carrier/ ppm	56	56	117.5	117.5
Time/ ms	0.9	0.7	1.0	1.0
Transfer II	CN-CP	CC DREAM	NC-CP	NC-CP
^{13}C field/ kHz	70	50	69	71
^{15}N field/ kHz	35	-	35	36
Shape	Tangent ^{13}C	Tangent ^{13}C	Tangent ^{13}C	Tangent ^{13}C
Carrier/ ppm	117.5	145	56	176
Time/ ms	15.0	7.0	16.0	18.0
Transfer III	NH-CP	CN-CP	CH-CP	CC DREAM
^{1}H field/ kHz	89	-	89	-
^{13}C field/ kHz	-	67	15	51
^{15}N field/ kHz	15	35	-	-
Shape	Tangent ^{1}H	Tangent ^{13}C	Tangent ^{1}H	Tangent ^{13}C
Carrier/ ppm	4.8	117.5	4.8	175
Time/ ms	1.2	16.0	0.7	9.5
Transfer IV	-	NH-CP	-	CH-CP
^{1}H field/ kHz	-	85	-	89
X field/ kHz	-	15	-	15
Shape	-	Tangent ^{1}H	-	Tangent ^{1}H
Carrier/ ppm	-	4.8	-	4.8
Time/ ms	-	1.0	-	0.7
t1 increments	86	104	48	42
Sweep width (t1)/ ppm	30	30	32	32
Acquisition time (t1)/ ms	6.7	8.1	8.7	7.6
t2 increments	48	48	120	120
Sweep width (t2)/ ppm	32	32	40	40
Acquisition time (t2)/ ms	8.7	8.7	7.0	7.0
t3 increments	1024	1024	1024	1024
Sweep width (t3)/ ppm	46.7	46.7	46.7	46.7
Acquisition time (t3)/ ms	12.9	12.9	12.9	12.9
^{1}H swfTPPM decoupling/ kHz	10	10	10	10
^{15}N WALTZ64 decoupling/ kHz	5	5	5	5
^{13}C WALTZ64 decoupling/ kHz	5	5	5	5
MISSISSIPPI wat.supp./ kHz	20	20	20	20
Interscan delay/ s	1.0	1.0	1.0	1.0
Number of scans	64	120	96	112
Measurement time/ h	89	198	181	187

**Experiment**	**hNH T_2'(^{1}H) (ADP:AlF_4^-:DNA)**
MAS frequency/ kHz	110
Field/ T	20
**Transfer I**	HN-CP
^{1}H field/ kHz	81
^{15}N field/ kHz	27
Shape	Tangent ^{1}H
Time / ms	1.1
**Transfer II**	NH-CP
^{1}H field/ kHz	76
^{15}N field/ kHz	27
Shape	Tangent ^{1}H
**T_2'(^{1}H) Measurement**	Hahn-Echo block
Relaxation delays / ms	0.001, 0.250, 0.500, 0.750, 1, 1.25, 1.5, 1.75
^{1}H carrier/ ppm	4.7
^{15}N carrier/ ppm	117.5
t1 increments	2048
Sweep width (t1)/ kHz	34.0
Acquisition time (t1)/ ms	30.0
t2 increments	512
Sweep width (t2)/ kHz	14.6
Acquisition time (t2)/ ms	17.5
Water Suppression	MISSISIPPI
^{1}H field / kHz	15
Time / ms	120
^{1}H swfttpm decoupling power/ kHz	10
^{1}H swfttpm decoupling pulse length/ µs	46
^{15}N WALTZ64 decoupling power/ kHz	15
^{15}N WALTZ64 decoupling pulse length/ µs	1.58
Interscan delay/ s	1
Number of scans	64
Measurement time/ h	86

References

[1] A. Bazin, M. V. Cherrier, I. Gutsche, J. Timmins, L. Terradot, *Nucleic Acids Res.* **2015**, *43*, 8564-8576.
[2] F. W. Studier, *Protein Expression and Purification* **2005**, *41*, 207-234.
[3] C. Gardiennet, A. K. Schütz, A. Hunkeler, B. Kunert, L. Terradot, A. Böckmann, B. H. Meier, *Angew. Chem. Int. Ed.* **2012**, *51*, 7855-7858.
[4] P. L. Gor'kov, R. Witter, E. Y. Chekmenev, F. Nozirov, R. Fu, W. W. Brey, *J. Magn. Reson.* **2007**, *189*, 182-189.
[5] A. Böckmann, C. Gardiennet, R. Verel, A. Hunkeler, A. Loquet, G. Pintacuda, L. Emsley, B. Meier, A. Lesage, *J. Biomol. NMR* **2009**, *45*, 319-327.
[6] R. Fogh, J. Ionides, E. Ulrich, W. Boucher, W. Vranken, J. P. Linge, M. Habeck, W. Rieping, T. N. Bhat, J. Westbrook, K. Henrick, G. Gilliland, H. Berman, J. Thornton, M. Nilges, J. Markley, E. Laue, *Nat Struct Mol Biol* **2002**, *9*, 416-418.
[7] W. F. Vranken, W. Boucher, T. J. Stevens, R. H. Fogh, A. Pajon, M. Llinas, E. L. Ulrich, J. L. Markley, J. Ionides, E. D. Laue, *Proteins: Structure, Function, and Bioinformatics* **2005**, *59*, 687-696.
[8] T. Stevens, R. Fogh, W. Boucher, V. Higman, F. Eisenmenger, B. Bardiaux, B.-J. van Rossum, H. Oschkinat, E. Laue, *J. Biomol. NMR* **2011**, *51*, 437-447.
[9] A. Abragam, *The Principles of Nuclear Magnetism*, Clarendon Press Oxford, **1961**.
[10] J. P. Yesinowski, H. Eckert, G. R. Rossman, *J. Am. Chem. Soc.* **1988**, *110*, 1367-1375.
[11] S. Penzel, A. Oss, M.-L. Org, A. Samoson, A. Böckmann, M. Ernst, B. H. Meier, *J. Biomol. NMR* **2019**.
[12] U. Sternberg, R. Witter, I. Kuprov, J. M. Lamley, A. Oss, J. R. Lewandowski, A. Samoson, *J. Magn. Reson.* **2018**, *291*, 32-39.
[13] K. Schmidt-Rohr, H. W. Spiess, in *Multidimensional Solid-State NMR and Polymers* (Eds.: K. Schmidt-Rohr, H. W. Spiess), Academic Press, San Diego, **1994**, pp. 69-134.
[14] A. A. Malär, S. Dong, G. Kehr, G. Erker, B. H. Meier, T. Wiegand, *ChemPhysChem* **2019**, *20*, 672-679.
[15] G. De Paëpe, N. Giraud, A. Lesage, P. Hodgkinson, A. Böckmann, L. Emsley, *J. Am. Chem. Soc.* **2003**, *125*, 13938-13939.
[16] D. Cala-De Paepe, J. Stanek, K. Jaudzems, K. Tars, L. B. Andreas, G. Pintacuda, *Solid State Nucl. Magn. Reson.* **2017**, *87*, 126-136.
[17] J. R. Lewandowski, J.-N. Dumez, Ü. Akbey, S. Lange, L. Emsley, H. Oschkinat, *J. Phys. Chem. Lett.* **2011**, *2*, 2205-2211.
[18] T. Wiegand, R. Cadalbert, D. Lacabanne, J. Timmins, L. Terradot, A. Bockmann, B. H. Meier, *Nat. Commun.* **2019**, *10*, 31.
